# Development of a Cation Exchange SPE-HILIC-MS/MS Method for the Determination of Ningnanmycin Residues in Tea and Chrysanthemum

**DOI:** 10.3390/foods13050635

**Published:** 2024-02-20

**Authors:** Aiping Li, Chen Wang, Zhenghao Wu, Yingying Liu, Zhenxia Hao, Chengyin Lu, Hongping Chen

**Affiliations:** 1 Tea Research Institute, Chinese Academy of Agricultural Sciences, Hangzhou 310008, Chinalchy@tricaas.com (C.L.); 2 Graduate School of Chinese Academy of Agricultural Sciences, Beijing 100081, China; 3 Laboratory of Quality and Safety Risk Assessment for Tea Products (Hangzhou), Ministry of Agriculture and Rural Affairs, Hangzhou 310008, China; 4 Key Laboratory of Tea Quality and Safety Control, Ministry of Agriculture and Rural Affairs, Hangzhou 310008, China

**Keywords:** ningnanmycin, tea, chrysanthemum, cation exchange, HILIC-MS/MS

## Abstract

Ningnanmycin is a widely used antibiotic in agricultural production that effectively controls fungal and viral diseases in tea trees and chrysanthemums. The polarity characteristic of ningnanmycin has posed limitations on the development of robust detection methods, thereby hindering effective monitoring and control measures. By combining cation exchange solid phase extraction (SPE) with hydrophilic interaction chromatography tandem mass spectrometry (HILIC-MS/MS), we have effectively tackled the issue pertaining to the separation and retention of ningnanmycin. The average recoveries of ningnanmycin in green tea, black tea, and chrysanthemum were 77.3–82.0%, 80.1–81.5%, and 74.0–80.0%, respectively. The intraday and interday relative standard deviations (RSDs) were below and equal to 7.7%. Good linearity was observed in the concentration range of 1–1000 μg/L (R2 > 0.998). The limits of detection (LODs) ranged from 1.1 μg/kg to 7.1 μg/kg, and the limits of quantification (LOQs) ranged from 3.6 μg/kg to 23.7 μg/kg for ningnanmycin. These results indicate the good accuracy, repeatability, reproducibility, and sensitivity of the method. It is suitable for detecting ningnanmycin in tea and chrysanthemum.

## 1. Introduction

Ningnanmycin is a cytosine nucleoside antibiotic widely used in agricultural production. It has proven effective against rice bacterial blight pathogens and tobacco mosaic virus [1]. Recent studies have also shown that ningnanmycin could effectively prevent and treat tea leaf spot, making it a valuable antiviral and antifungal agent [2].

However, it is important to note that ningnanmycin has subchronic toxicity to mammals. Studies have shown that it led to changes in indicators such as the inhibition of weight gain and the reduction in organ weight in rats. To ensure the safety of its use, China has established an acceptable daily intake (ADI) value for ningnanmycin at 0.24 mg/kg·bw·d [3].Currently, there is limited research on the detection methods for ningnanmycin residues in crops. The limited information available on ningnanmycin may result in the improper use of this antibiotic, which is a matter of concern. Inadequate supervision and control of ningnanmycin in crops could potentially jeopardize human health, underscoring the significance of stringent oversight.

**Figure 1:**
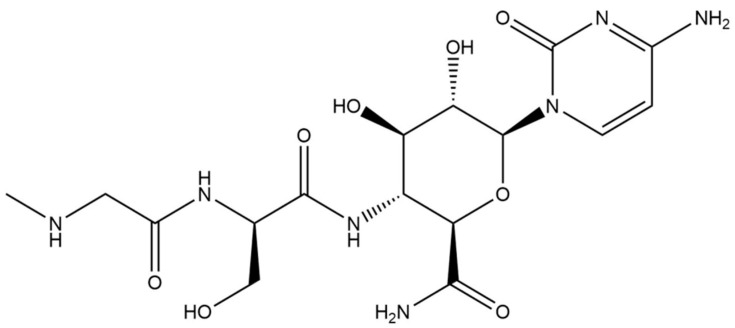
Chemical structure of ningnanmycin.

In order to ensure consumer safety, it is necessary to establish a sensitive and specific analysis method for detecting residues of ningnanmycin. As shown in Figure 1, ningnanmycin is highly polar and contains a large number of amino groups and hydroxyl groups, which are difficult to retain using traditional reversed-phase liquid chromatography (RPLC). Consequently, ningnanmycin co-elutes with other highly polar substances in the matrix, making it difficult to accurately quantitate. This co-elution negatively impacts the precision and accuracy [4]. To overcome these challenges, the hydrophilic interaction liquid chromatography (HILIC) mode serves as an efficient method for retaining and separating polar analytes. The HILIC mode utilizes a gradient elution system with a mobile phase of high acetonitrile and low water content, along with a polar stationary phase. This mode allows for the separation of analytes in order of increasing hydrophilicity (polarity) [5]. Furthermore, the use of high organic solvents in the mobile phase could enhance the sensitivity of MS [6]. Consequently, the HILIC mode proves to be exceptionally effective in the separation of polar and hydrophilic small molecules, carbohydrates, glycans, and peptides. It is especially well-suited for the quantitative analysis and detection of ningnanmycin.

Current research on the analysis of ningnanmycin residues primarily focuses on simple matrices such as rice, fruits, and vegetables. The method developed by Li et al. to detect ningnanmycin residues in green tea leaves has a limit of detection (LOD) of 15 μg/kg and a limit of quantification (LOQ) of 50 μg/kg. There is no method for detecting ningnanmycin residues in black tea and chrysanthemums [7,8]. Thus, a sensitive method for the determination of ningnanmycin in tea and chrysanthemums is needed. However, tea and chrysanthemum contain complex components such as polyphenols, polysaccharides, pigments, and alkaloids, which could strongly affect the matrix effect in LC-MS/MS, leading to compromised detection sensitivity and potential instrument contamination [9]. Therefore, effective pretreatment for purification is crucial.

Solid-phase extraction (SPE) is a widely used method for purification in the detection and analysis of pesticide residues in agricultural products [10,11]. There are several types of SPE cartridges available, such as strong cation exchange cartridges (SCX) and polymer cation exchange cartridges (PCX) [12]. In comparison to SCX cartridges, PCX cartridges have the advantage of combining reversed phase and cation exchange properties. This enables them to simultaneously separate neutral, acidic, and basic compounds. On the other hand, they can be drained and are easy to operate [13,14], making them suitable for the efficient extraction of amino-containing ningnanmycin in tea and chrysanthemum. 

Therefore, this study aimed to develop a detection method for ningnanmycin in tea and chrysanthemum. The method involved methanol/water vortex extraction, followed by PCX SPE cartridge purification and finally HILIC-MS/MS for quantitative analysis. The successful implementation of this method will offer valuable technical support for the monitoring and supervision of ningnanmycin levels in tea and chrysanthemum.

## 2. Materials and Methods

### 2.1. Analytical Instruments

The instrumentation employed includes the AB SCIEX TQ 5500 tandem triple quadrupole mass spectrometer (AB SCIEX, Framingham, MA, USA), the LC-30A ultra-high performance liquid chromatograph (Shimadzu, Japan), the Milli-Q ultrapure water system (Millipore, Milford, MA, USA), and the SIGMA 4–16 KS high-speed refrigerated centrifuge (SIGMA, Osterode, Germany).

### 2.2. Reagents and Materials

Standard ningnanmycin (CAS: 156410-09-2, purity 99.1%) was purchased from First Standard (Tianjin, China). Chromatographic grade methanol and acetonitrile were purchased from Thermo Fisher Scientific (Waltham, MA, USA). Analytical-grade ammonium hydroxide was purchased from Shanghai Aladdin Biochemical Technology Co., Ltd., (Shanghai, China). Bond Elut PCX solid phase extraction cartridges (500 mg/6 mL) were purchased from Agilent Technologies, USA. The nylon filter membrane (13 mm × 0.22 μm) used to filter the extract was provided by Shanghai Anpu Experimental Technology Co., Ltd. (Shanghai, China).

### 2.3. Preparation of the Standard Solution

The standard ningnanmycin solution was meticulously measured at 1 mL, with a concentration of 1000 μg/mL. Following this, a standard stock solution was prepared with a concentration of 100 μg/mL, using methanol as the solvent. It is crucial to store this solution in the dark at −20 °C. The blank tea extract was employed to create a matrix standard solution, facilitating the quantitative analysis of ningnanmycin.

### 2.4. Sample Pretreatment

Tea (1.00 g) was carefully weighed and placed into a 50 mL centrifuge tube. Subsequently, 5 mL of methanol/water (20/80, *v*/*v*) was added to the tube, followed by vortexing at a speed of 2500 rpm for a duration of 10 min. The resulting mixture was then subjected to centrifugation at a speed of 10000 rpm for 5 min in order to achieve phase separation. Following centrifugation, the supernatant was carefully collected, and an additional 5 mL of methanol/water (20/80, *v*/*v*) was added to the tube. This extraction process was repeated, and the combined supernatant was transferred to a 15 mL centrifuge tube to undergo purification.

**Figure 2:**
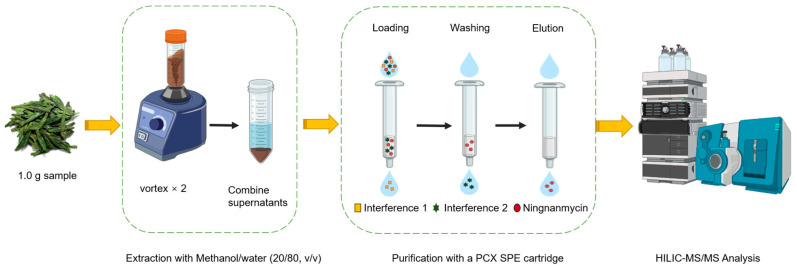
Schematic diagram of sample pretreatment.

The PCX SPE cartridge was activated by initially using 5 mL of methanol, followed by 5 mL of methanol/water (20/80, *v*/*v*) solution. Subsequently, all collected solutions were transferred to the PCX SPE purification cartridge. A washing step was then performed using 5 mL of water and 5 mL of methanol. Finally, the cartridge was eluted with a 10 mL solution of methanol containing 10% ammonium hydroxide. The eluate was collected, injected into a 1.5 mL sample vial through a 0.22 μm polytetrafluoroethylene filter, and subjected to analysis using UHPLC-MS/MS. As shown on Figure 2.

### 2.5. Instrumental Conditions

Liquid chromatographic conditions: The chromatographic column used was an Agilent Poroshell 120 HILIC column (150 mm × 2.1 mm, 1.9 μm). The mobile phases consisted of water containing 50 mmol/L ammonium formate (mobile phase B) and acetonitrile (mobile phase A). The gradient elution program was as follows: 0–1 min, 5% A; 1–8 min, 5% to 60% A; 8–11 min, 60% A; 11–11.1 min, 60% to 5% A; 11.1–15 min, 5%A. The flow rate was set at 0.3 mL/min. The column temperature was maintained at 40 °C. The injection volume used was 3 μL.

Mass spectrometry conditions: Analysis was performed using a positive mode electrospray ion source in positive mode (ESI^+^). The ion source temperature was set at 500 °C, while the curtain gas and collision gas pressures were maintained at 35 psi and 7 psi, respectively. The spray voltage was 4500 V. The atomization gas pressure and auxiliary gas pressure were both set at 50 psi.

### 2.6. Matrix Effect

The evaluation of the matrix effect (ME) involves comparing the slope of the matrix-matched standard curve with the solvent calibration curve. The ME value provides information about the degree of influence that the matrix has on the signal. A positive ME value (ME > 0) indicates signal amplification resulting from the matrix, whereas a negative ME value (ME < 0) suggests signal suppression caused by the matrix [15,16]. In Equation (1), A represents the slope of the matrix-matched calibration curve, while B represents the slope of the solvent calibration curve.

(1) $$ME=\left(\frac{A}{B}-1\right)\times 100\%$$

## 3. Results and Discussion

### 3.1. Optimization of Instrument Parameters

#### 3.1.1. Optimization of MS/MS Parameters

The optimization of the instrument parameters can have a profound impact on the sensitivity, selectivity, accuracy, and reproducibility of an analysis, thus guaranteeing the quality and reliability of the acquired test data [17].

To identify the optimal spectral conditions, we performed direct injection of the standard ningnanmycin into the mass spectrometer for analysis of the standard solution. Ningnanmycin contains five amino groups, four carbonyl groups, and three hydroxyl groups, which readily form protonated ions in the ESI^+^ ionization source. Therefore, we chose the positive ion mode for our analysis. In this mode, a full-scan analysis was conducted, recording the MS spectrum in the m/z range of 50 to 500 in order to obtain the precursor ions of the analyte. The protonated ion [M + H]^+^ of the analyte showed the strongest signal with an m/z value of 444.1. Subsequently, product ion scans were performed, selecting the two most abundant ions for each analyte for final qualitative and quantitative analysis. The MRM channel parameters were optimized to achieve the best response, typically selecting the most abundant ion for quantification. However, we observed that the response of ningnanmycin at m/z 315.1 was the highest, leading us to choose 315.1 as the quantification ion. The final MRM mass spectrometry parameters are provided in Table S1.

Based on the triple quadrupole product ion scan spectrum, we deduced the mass spectral fragmentation pathway of ningnanmycin, as shown in Figure S1. The protonated molecular ion of ningnanmycin ([M + H]^+^, m/z 444.17) underwent cross-ring cleavage, losing +C_4_H_5_N_3_O and rearranging to yield the product ion at m/z 333.13. Through a β-elimination reaction, the primary product ion at m/z 315.12 was generated by the facile loss of water from the hydroxyl group on the side chain. Breakage of the amide bond at m/z 315.12, with the loss of −C_3_H_5_NO, produced the product ion at m/z 244.09.

#### 3.1.2. Optimization of Chromatographic Conditions

Optimizing the chromatographic conditions not only enhances the detection performance but also mitigates the influence of interfering substances [18].

**Figure 3:**
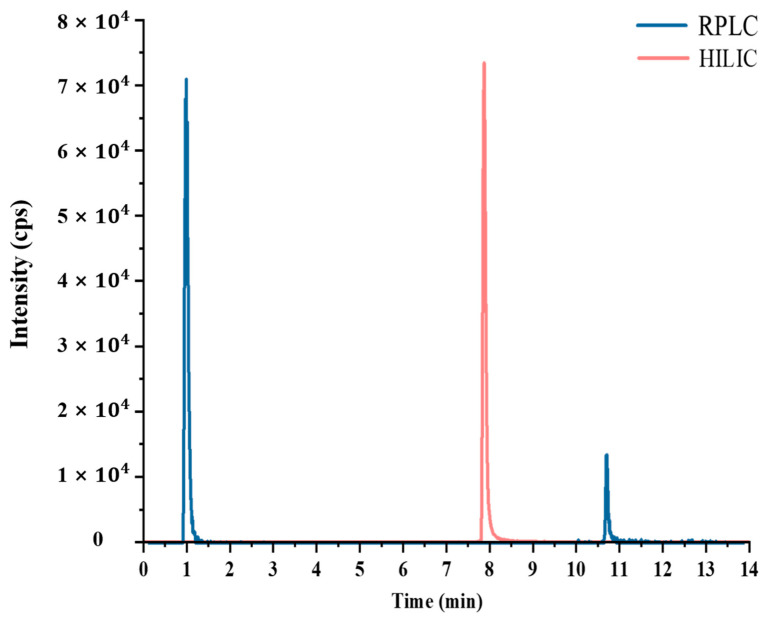
The chromatogram of ningnanmycin was determined at a concentration of 0.1 μg/mL using UPLC-MS/MS combined with an HILIC column/RPLC column.

In order to optimize the chromatographic separation mode, we selected two common modes: RPLC and HILIC [19]. For this study, we chose the Waters Acquity UPLC HSS T3 (100 nm × 2.1 mm, 1.8 μm) RP chromatographic column and the Agilent Poroshell 120 (150 mm × 2.1 mm, 1.9 μm) HILIC chromatographic column for optimization. Figure 3 displays the chromatograms of ningnanmycin obtained from these two columns. On the RP chromatographic column, ningnanmycin had a retention time of 1.02 min, indicating almost no retention as it flowed out with the mobile phase that initially contained a high proportion of water. On the HILIC column, however, ningnanmycin displayed optimal retention with a retention time of 7.95 min. This is because ningnanmycin has a highly polar nature, as evidenced by its n-octanol-water partition coefficient of −5.79. Analytes with high polarity exhibit a low affinity for the non-polar C_18_ stationary phase commonly used in reversed-phase (RP) chromatography. Consequently, strongly polar compounds tend to elute with the highly water-soluble mobile phase after passing through the column, making it challenging to retain them within the chromatographic column for an extended period [20,21]. To address this issue, the Agilent Poroshell 120 HILIC chromatographic column was employed, utilizing silica gel as the stationary phase. In the HILIC chromatographic column, the mixture of organic phase and water facilitates the formation of free silanol groups, generating a negatively charged acidic surface. This, in turn, promotes interaction with polar analytes and prolongs their retention time on the column [22]. The disparity in the retention time of the compound on the chromatographic column will have an impact on the sensitivity of the compound in HPLC-MS/MS. A shorter retention time will result in co-elution with other highly polar matrix components, leading to matrix effects and a significant reduction in sensitivity [23].

**Figure 4:**
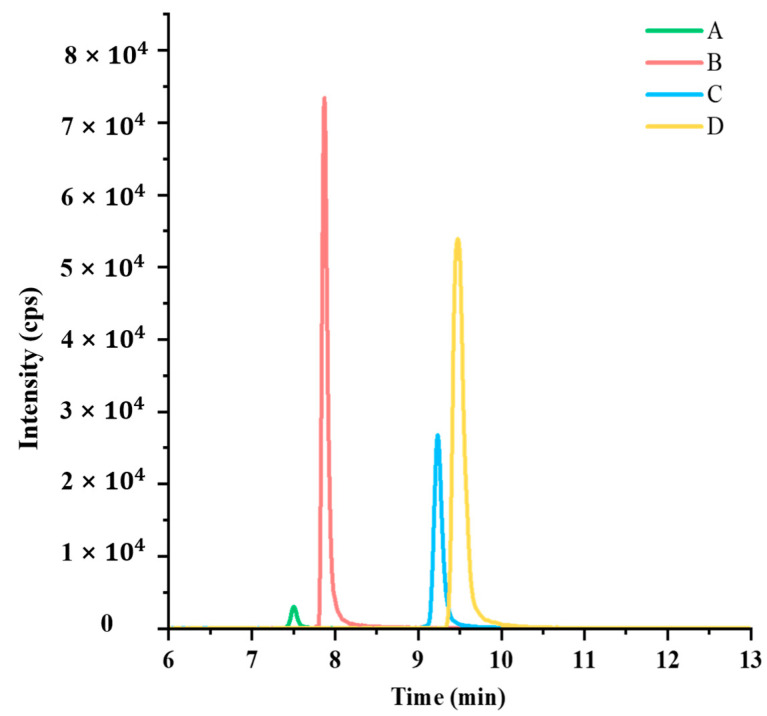
Chromatograms of ningnanmycin in four chromatographic columns at a concentration of 0.1 μg/mL. Note: (**A**) Merck SeQuant ZIC-HILIC (150 mm × 2.1 mm, 3.5 μm), (**B**) Agilent Poroshell 120 HILIC (150 mm × 2.1 mm, 1.9 μm), (**C**) Waters ACQUITY UPLC BEH Amide (100 mm × 2.1 mm, 1.7 μm), (**D**) Agilent Polaris NH_2_ HPLC (150 mm × 2.0 mm, 3.0 μm).

Taking these factors into consideration, the HILIC chromatographic column was the most suitable choice for detecting ningnanmycin. The response signal of a compound is determined by the hydrophilicity and functional groups of the stationary phase [24]. In this study, four HILIC columns were selected, each with different properties: underivatized silica gel, amino-containing silica gel, zwitterionic, and amide bonded phases. The response signals and peak shapes of ningnanmycin were compared among these four columns under the same conditions. The results, as shown in Figure 4, indicate that the retention times for all four chromatographic columns fell within the range of 6 to 10 min. Notably, when the Agilent Poroshell 120 HILIC column (150 mm × 2.1 mm, 1.9 μm) was employed for the detection of ningnanmycin, it exhibited the highest response signal of 7.3 × 10^4^ cps, surpassing the other three columns. Additionally, this column demonstrated the narrowest peak width and a favorable peak shape.

**Figure 5:**
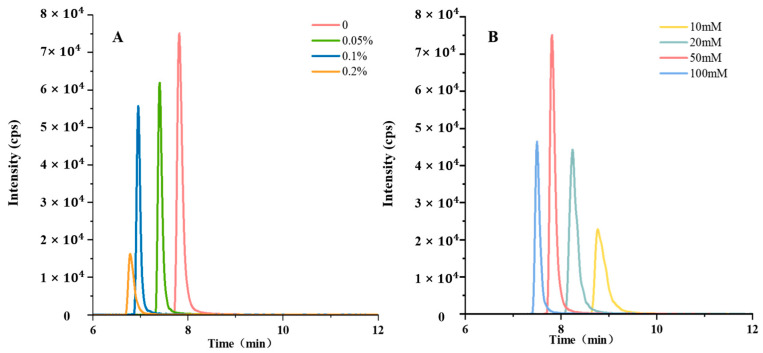
Chromatograms of ningnanmycin under different mobile phase conditions at a concentration of 0.1 μg/mL. (**A**) Formic acid, (**B**) ammonium formate.

In addition to factors related to the chromatographic column, the response signal of the compound is also influenced by the mobile phase. Modifying the pH value of the mobile phase or incorporating a buffer could enhance the ionization efficiency of the analyte, optimize the shape of the peaks, and increase the sensitivity [25]. When utilizing acetonitrile as the organic phase, formic acid and ammonium formate were added separately to the water phase. The objective was to assess the response signal and peak shape of ningnanmycin under varying conditions. The obtained results are illustrated in Figure 5. When varying concentrations of formic acid were introduced into water, it became evident that the response signal of ningnanmycin diminished as the concentration of formic acid increased. The most robust response signal was obtained when ningnanmycin was free of formic acid. Specifically, the introduction of 0.05% formic acid resulted in a 17.7% signal decrease, while 0.2% formic acid caused a more pronounced 78.4% reduction in the signal. To optimize the peak shape, ammonium formate was added to the aqueous phase. Our observations demonstrated that ningnanmycin exhibited a more favorable peak shape under the conditions of the mobile phase containing 50 mmol/L ammonium formate. Consequently, the aqueous phase was chosen to contain a 50 mmol/L ammonium formate solution. 

### 3.2. Optimization of Sample Preparation

#### 3.2.1. Optimization of Extraction Conditions

In tea pesticide testing, the samples are exceedingly intricate and comprise a multitude of interfering substances. Employing suitable extraction conditions could enhance the extraction efficiency and mitigate the presence of interfering substances [26]. In this section, the optimization of the extraction solvent and method was conducted. The C_18_ SPE cartridge was chosen as the purification technique for pretreating the matrix standard sample. The optimal extraction conditions were determined by comparing the extraction efficiency achieved under various conditions. The extraction rate was calculated by dividing the total recovery rate by the recovery rate obtained during the purification step.

**Figure 6:**
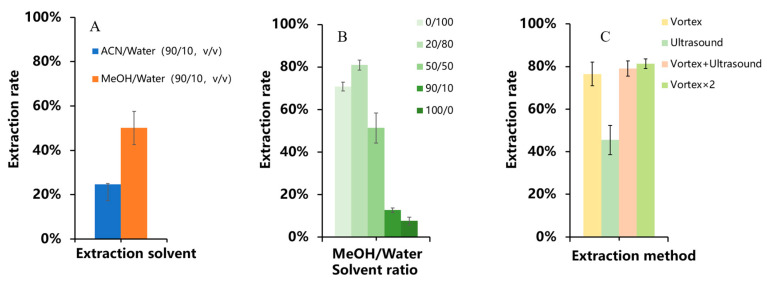
Extraction rate of ningnanmycin under different extraction conditions. (**A**) Extraction solvent type, (**B**) V _m_/V _w_ in the extraction solvent, (**C**) extraction method (*n* = 3).

The results are depicted in Figure 6. The extraction efficiency of a methanol/water (90/10, *v*/*v*) solution was determined to be 50.08%, whereas the extraction of an acetonitrile/water (90/10, *v*/*v*) solution was found to be 24.46%. In accordance with the principle of miscibility, it could be deduced that the solubility of the highly polar ningnanmycin compound was higher in the highly polar methanol solvent compared to in acetonitrile [27]. When examining the effect of different proportions of methanol solutions on the extraction, it was observed that the highest extraction of ningnanmycin was achieved in a solution containing methanol/water (20/80, *v*/*v*). This could be attributed to the highly polar nature of ningnanmycin and its excellent solubility in water. Additionally, a certain concentration of methanol aids in the separation of ningnanmycin 1 the matrix.

In terms of optimizing the extraction method, the results indicated that the vortex extraction yielded a good extraction efficiency for ningnanmycin, while the efficiency of the ultrasonic extraction was significantly lower. Furthermore, increasing the number of vortexing cycles improved the extraction of ningnanmycin. The final extraction conditions were determined to be vortex extraction using a 5 mL solution of methanol and water (20/80, *v*/*v*) for a total of two cycles, with each cycle lasting 10 min.

#### 3.2.2. Optimization of the Purification Conditions

**Figure 7:**
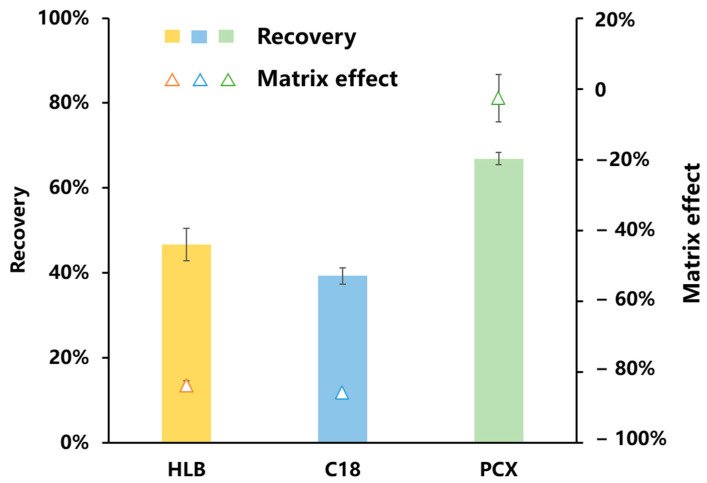
Recovery and matrix effects of ningnanmycin under different SPE conditions (*n* = 3).

When optimizing the purification conditions, it is crucial to optimize the type, specifications, and working solvent of the solid-phase extraction (SPE) cartridge. In this study, three different SPE cartridges with distinct stationary phases were chosen, namely the polymer cation exchange cartridge (PCX), the hydrophilic–lipophilic balance cartridge (HLB), and the reversed-phase cartridge (C_18_). The obtained results are presented in Figure 7. The highest recovery rate of ningnanmycin, achieved using the PCX SPE cartridge, was 66.91%, surpassing the recovery rates of 46.6% and 39.26% obtained using the HLB SPE and C_18_ SPE cartridges, respectively. Furthermore, the PCX SPE cartridge demonstrated a matrix effect of −5%. Similar to the research findings of Li et al., it can be observed that the inhibitory effect of the HLB cartridge on the matrix was more pronounced compared to that of the cation exchange cartridge [8]. Considering the combined purification effect, recovery rate, and matrix effect, the PCX SPE cartridge emerged as the optimal choice for the purification of ningnanmycin in tea.

**Figure 8:**
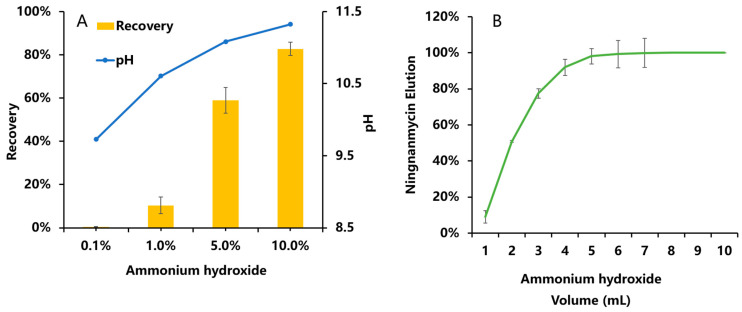
Recovery rate of ningnanmycin in (**A**) different proportions of ammonium hydroxide and methanol eluent and pH; (**B**) elution of different volumes (*n* = 3).

The ammonium hydroxide content and elution volume of the methanol eluate were optimized. In this study, ammonium hydroxide was used to adjust the pH of the methanol eluent, and the elution effects of different pH values on ningnanmycin were observed. Figure 8A shows the results. A higher concentration of ammonium hydroxide resulted in a higher recovery rate. A good recovery rate of 82.7% was achieved for ningnanmycin in the methanol eluent containing 10% ammonium hydroxide. This phenomenon occurs due to the employment of methanol containing ammonium hydroxide during elution. NH_4_^+^ can associate with the anionic groups on the stationary phase via electrostatic attraction, consequently displacing the initially bound ningnanmycin [28]. In contrast to prior research, this method requires only a single pass through the SPE cartridge, rendering it highly user-friendly and straightforward to execute.

To ensure the thorough elution of ningnanmycin, an examination of its elution curve was conducted, as depicted in Figure 8B. It was observed that at an elution volume of 6 mL, 99.28% of ningnanmycin was successfully eluted. Additionally, no significant response was recorded at an elution volume of 10 mL, suggesting a complete elution rate of 100%. These findings suggest that ningnanmycin was entirely eluted from the PCX SPE cartridge. Consequently, an eluent consisting of 10 mL of methanol with a 10% concentration of ammonium hydroxide was chosen.

**Figure 9:**
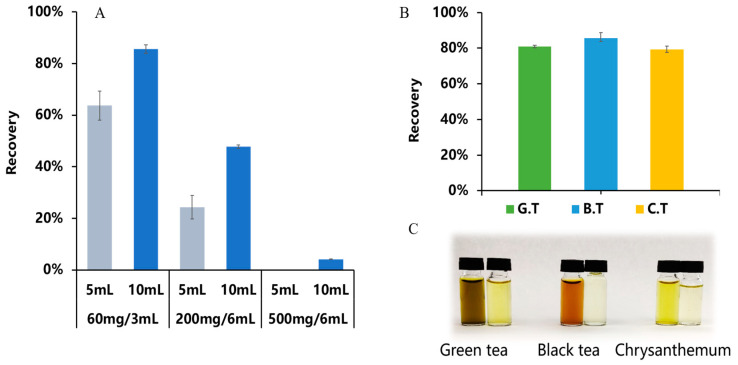
(**A**) The recovery in black tea under various PCX SPE cartridge conditions, (**B**) purification efficiency in three matrices, and (**C**) recovery rates of ningnanmycin. Note: G.T means green tea, B.T means black tea, and C.T means chrysanthemum (*n* = 3).

Finally, we conducted an investigation into the influence of the PCX SPE cartridges with varying specifications on the recovery rate of ningnanmycin in the black tea matrix. The findings are displayed in Figure 9A. Under the same elution volume, the recovery rate of ningnanmycin exhibited a gradual decline as the PCX SPE cartridge specifications expanded. This phenomenon could be attributed to the increased capacities and surface areas of larger SPE cartridges, allowing for greater accommodation of analytes and adsorbed materials. However, when the elution volume remained constant, incomplete elution of the target substances occurred, leading to a subsequent decrease in the recovery rate [29]. To enhance the recovery rate, it may be necessary to increase the elution volume proportionally to the cartridge size. Nevertheless, it is challenging to collect eluates larger than 20 mL. Therefore, we selected a PCX SPE cartridge with a specification of 60 mg/3 mL. After eluting with 10 mL of methanol containing 10% ammonium hydroxide, the recovery rate reached 85.5%. We then applied this method to detect ningnanmycin in green tea and chrysanthemum matrices. The results, illustrated in Figure 9B, indicated a recovery rate of 80.8% in green tea and 79.3% in chrysanthemum. 

Matrix suppression or enhancement effects could have an impact on the accuracy of quantitative analysis using LC-MS/MS [30]. The matrix effect primarily arises from the endogenous components present in biological samples, which persist in the extraction solution after pretreatment. Such components include ionic particulate matter components (electrolytes, salts), highly polar compounds (phenols, pigments), and diverse organic compounds (saccharides, amines, urea, and analogues of the target substances being analyzed) [31,32]. This method demonstrated proficient pigment removal capabilities in all three matrices. Following the purification process, the color of the extraction solution noticeably lightened, thereby decreasing contamination to the instrument. This effect is visually demonstrated in Figure 9C.

The pretreatment process involved the utilization of a total volume of 23 mL of organic solvent. Prior studies investigating the detection of ningnanmycin in tea required the use of methylene chloride and multiple purification columns in order to achieve a satisfactory recovery rate, resulting in a cumulative consumption of 40 mL of solvent [8]. However, the present study proposes a more sustainable approach by employing a PCX solid-phase extraction cartridge and optimizing the elution conditions. This novel method not only improves the recovery rate in an environmentally friendly method but also streamlines and expedites the pretreatment process.

### 3.3. Method Validation

#### 3.3.1. Accuracy and Precision

Three different concentrations of ningnanmycin were utilized in the spiked recovery experiments conducted on blank samples to ascertain the method’s accuracy and precision. The outcomes are presented in Table 1. The recovery rates of ningnanmycin in green tea, black tea, and chrysanthemum were approximately 77.3% to 82.0%, 80.1% to 81.5%, and 74.0% to 80.0%, respectively, indicating a high level of accuracy. Additionally, the interday RSDs ≤ 7.7%, while the intraday RSDs ≤ 5.9%, demonstrating excellent repeatability and reproducibility.

#### 3.3.2. Sensitivity

The sensitivity of the method is assessed through the determination of the limit of quantification (LOQ) and the limit of detection (LOD) [33,34]. We utilized matrix matching calibration methodology to ascertain the limits of detection (LOD) and quantification (LOQ). We compared the sample’s concentration against the corresponding concentration of the blank test. We recorded the measured signal intensity (S) and noise intensity (N) of the tested sample. The LOD is defined as the lowest concentration of the sample at which the signal-to-noise ratio (S/N) reaches 3, while the LOQ refers to the lowest concentration at which the S/N ratio reaches 10. This method was the first attempt to quantify ningnanmycin levels in black tea and chrysanthemum. As indicated in Table 2, the detection limit for ningnanmycin in the green tea, black tea, and chrysanthemum matrices ranged from 1.1 to 7.1 μg/kg. The LOQs for ningnanmycin in green tea, black tea, and chrysanthemum were found to be 3.6 μg/kg, 11.3 μg/kg, and 23.7 μg/kg, respectively. By conducting a comparative analysis of the previous research findings, as presented in Table S2, it is evident that the results obtained demonstrate a significantly lower LOD and LOQ for ningnanmycin in green tea compared to those reported in earlier studies.

#### 3.3.3. Linearity and Matrix Effect

To establish the linear quantitative correlation between various concentrations of ningnanmycin and the response of the instrument, a series of six concentrations (ranging from 1 to 1000 μg/L) of ningnanmycin matrix matching standard solutions were prepared in different tea matrices. Subsequently, a standard curve was constructed. Measurements were conducted in triplicate, and the least squares method was applied to process the data and derive the linear equations and correlation coefficients. The obtained results are displayed in Table 2. Within the concentration range of 1 to 1000 μg/L, the coefficient of determination (R^2^) for the ningnanmycin standard curve exceeded 0.998, indicating a strong linear relationship.

The impact of the matrix effect was assessed by comparing the slopes of the standard curves, which were prepared using a blank matrix-matched standard solution and a standard solution prepared with pure solvent. It is important to note that the same pesticide may exhibit distinct matrix effects in different types of tea [35]. As presented in Table 2, the percentages of ningnanmycin in green tea, black tea, and chrysanthemum were 18.9%, 1.6%, and 23.8%, respectively. Notably, green tea and chrysanthemum displayed a weak matrix effect, thus necessitating the use of matrix matching standard solutions for quantification purposes.

## 4. Conclusions

A robust, simple, and sensitive method for the precise quantification of ningnanmycin in tea and chrysanthemum samples was established and validated in this study, using cation exchange SPE-HILIC-MS/MS. The extraction of ningnanmycin from the sample involved two rounds of vortexing using a methanol/water (20/80, *v*/*v*) solution. Subsequently, the extract was purified using a 60 mg/ 3 mL PCX SPE cartridge, and the elution of ningnanmycin was accomplished using a 10 mL solution containing 10% ammonium hydroxide in methanol. Finally, HILIC-MS/MS was utilized for quantitative analysis. This method exhibits the following characteristics: (1) good accuracy and precision, with average recovery rates of 77.3–82.0% for ningnanmycin in green tea, 80.1–81.5% for black tea, and 74.0–80.0% for chrysanthemum, and the intraday and interday precisions were below 7.7%; (2) high sensitivity, with limits of detection (LODs) for ningnanmycin and chrysanthemum ranging from 1.1 to 7.1 μg/kg and limits of quantification (LOQs) ranging from 3.6 to 23.7 μg/kg; (3) a wide linear range, with a well-established linear relationship (R² > 0.998) within the concentration range of 1–1000 μg/L. Overall, this method exhibits high sensitivity and precision, rendering it suitable for the detection of ningnanmycin residues in green tea, black tea, and chrysanthemum. Therefore, it could offer valuable technical support for their monitoring and supervision.

## Figures and Tables

**Table 1:** Recovery and precision of ningnanmycin in three matrices (n = 5).

Tea Samples	Recoveries (%)			Interday Precision (RSD, %)			Intraday Precision (RSD, %)		
	Spiked Level (μg/kg)			Spiked Level (μg/kg)			Spiked Level (μg/kg)		
	C1	C2	C3	C1	C2	C3	C1	C2	C3
G.T	77.3	78.4	82.0	4.9	6.9	3.2	4.2	5.1	0.5
B.T	80.1	80.5	81.5	3.9	7.0	3.6	1.0	0.6	0.7
C.T	74.0	80.0	75.3	5.8	7.7	4.1	2.5	1.5	0.6

Note: In the case of ningnanmycin, C1, C2, and C3 were spiked at levels of 10, 50, and 100 μg/kg in black tea and green tea and at levels of 50, 100, and 500 μg/kg in chrysanthemum. G.T means green tea, B.T means black tea, and C.T means chrysanthemum

**Table 2:** Linearity, equation, ME, LODs, and LOQs of ningnanmycin (*n* = 5).

Tea Samples	Calibration Equation	R^2^	Linear Range	LOQ	LOD	ME
			(μg/L)	(μg/kg)	(μg/kg)	(%)
G.T	y = 706.06x + 1055.7	0.9998	1–1000	3.6	1.1	18.9
B.T	y = 621.24x + 1496.5	0.9995	1–1000	11.3	3.4	1.6
C.T	y = 735.08x + 1418.1	0.9985	1–1000	23.7	7.1	23.8

Note: G.T means green tea, B.T means black tea, and C.T means chrysanthemum.

## Data Availability

The original contributions presented in the study are included in the article/Supplementary Material, further inquiries can be directed to the corresponding author.

## Supplementary Materials

The following supporting information can be downloaded at: https://www.mdpi.com/article/10.3390/foods13050635/s1, Figure S1: Secondary mass spectrum and mass spectrum fragmentation pathway of the [M+H]+ion of Ningnanmycin; Table S1: Mass Spectrometric Optimization Parameters of ningnanmycin in Multiple Reaction Monitoring Mode (MRM); Table S2: Comparison of our method with previous methods for the determination of ningnanmycin residues.
