# Supplementary material for: Development of a Cation Exchange SPE-HILIC-MS/MS Method for the Determination of Ningnanmycin Residues in Tea and Chrysanthemum
Source: Foods. 2024 Feb 20;13(5):635. doi: 10.3390/foods13050635 (PMC10930893; doi:10.3390/foods13050635)
Supplement: Supplementary file 1 [file foods-13-00635-s001.zip › foods-2842570-supplementary.pdf]

Table S1. Mass Spectrometric Optimization Parameters of ningnanmycin in Multiple Reaction Monitoring Mode (MRM).

Compounds	Molecular Formula	Precursor ion (m/z)	Product ions (m/z)	Collision energy (V)	Decluster potential (V)	Retention Time (min)
**NNM**	$C_{16}H_{25}O_8N_7$	444.1	315.4*/333.0	25/71	101	9.10

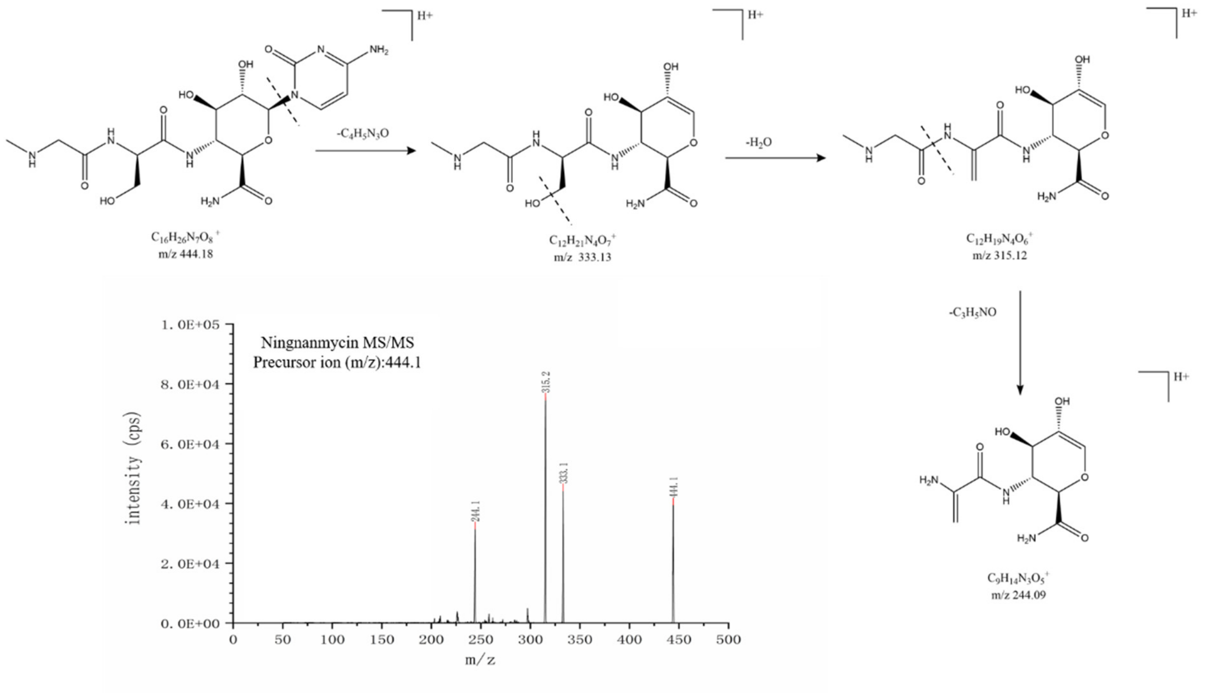

Figure S1. Secondary mass spectrum and mass spectrum fragmentation pathway of the $[M+H]^+$ion of Ningnanmycin.

Table S2 Comparison of our method with previous methods for the determination of ningnanmycin residues

Pretreatment methods	Main extraction solvent	Analyzed Matrix	Organic usage (mL)	Methods	Linear Range(μg/L)	LOD (μg/kg)	LOQ (μg/kg)	Recovery(%)	RSD(%)	References
HLB-MCX	Dichloromethane+water	Geen tea	40	UPLC-MS/MS	2-200	15	50	75-77	< 9	[8]
PCX	methanol/water (20/80, v/v)	Geen tea	23	UPLC-MS/MS	1-1000	1.1	3.6	77.3-82.0	< 6.9	This work
		Black tea				3.4	11.3	80.1-81.5	< 7	
		Chrysanthemum				7.1	23.7	74.0-80.0	< 7.7	
